# Stress, coping, protective factors, and quality of life in parents of infants with CHD: associations with state anxiety

**DOI:** 10.1017/S1047951126111809

**Published:** 2026-03

**Authors:** Jessica Bainton, Felicia L. Trachtenberg, D’Andrea Freemon, Michael A. Fremed, Emily Klingman, Linda M. Lambert, Andrew W. McCrary, Brian W. McCrindle, Kathleen Rathge, Anjali Sadhwani, David E. Segar, Rachel J. Shustak, Erica Sood, Karen Uzark, Jodie K. Votava-Smith, Frances Woodard Kline, Kathleen A. Mussatto

**Affiliations:** 1 Division of Cardiology, The Hospital for Sick Children, Canada; 2 Carelon Research Inc, USA; 3 National Heart Lung and Blood Institute, USA; 4 Division of Pediatric Cardiology, Department of Pediatrics, Columbia University Irving Medical Center, USA; 5 Division of Pediatric Cardiology, Department of Pediatrics, NewYork-Presbyterian Morgan Stanley Children’s Hospital, USA; 6 Children’s Healthcare of Atlanta Inc, USA; 7 Cardiothoracic surgery, University of Utah Health, USA; 8 Pediatrics, Duke University Hospital, USA; 9 Heart Institute, Cincinnati Children’s Hospital Medical Center, USA; 10 Boston Children’s Hospital, USA; 11 Children’s Wisconsin Herma Heart Institute, USA; 12 Division of Cardiology, The Children’s Hospital of Philadelphia, USA; 13 Cardiology, Nemours Children’s Hospital Delaware, USA; 14 Pediatric Cardiology, University of Michigan Mott Children’s Hospital, USA; 15 Children’s Hospital Los Angeles, USA; 16 Department of Pediatric Cardiology, Medical University of South Carolina, USA; 17 School of Nursing, Milwaukee School of Engineering, USA; 18 Children’s Hospital of Wisconsin Inc, USA

**Keywords:** CHD, Norwood, parents, anxiety, stress, coping

## Abstract

**Introduction:**

The Family Adaptation study, ancillary to the Single Ventricle Reconstruction Trial, examined the prevalence of anxiety and its associations with stress, psychosocial factors, and quality of life measures in parents of infants who underwent the Norwood procedure.

**Materials and methods:**

Two hundred and fifteen parents (143 mothers and 72 fathers) of 146 infants completed state anxiety (State Anxiety Inventory), stress, psychosocial, and quality of life measures post-Norwood, post-Stage II, and at a final visit (median child age: 14 months).

**Results:**

A substantial proportion of parents reported severe anxiety symptoms following the Norwood surgery, with 61% of mothers and 43% of fathers affected, decreasing over time to 46% and 33% by the final visit, respectively. Mothers’ average STAI-S scores were significantly higher than fathers’ post-Norwood (47.7 ± 13.2 versus 43.5 ± 11.8, *p* = 0.03), declining to 42.1 ± 13.0 versus 39.0 ± 9.6 (*p* = 0.14) at the final visit. Stress related to parenting a child with a serious illness was a stronger and more consistent predictor of mothers’ anxiety over time (highest *R* ^2^ = 0.49 for emotional distress), whereas insufficient coping and fewer protective factors were greater and more consistent predictors for fathers (highest *R* ^2^ = 0.40 for mastery and health). Quality of life was a consistent predictor of state anxiety for both mothers and fathers.

**Conclusion:**

Anxiety is elevated in parents of infants who underwent the Norwood procedure and is influenced by a complex interplay of stress, psychosocial factors, and quality of life. Addressing these factors is crucial for improving parents’ mental health, which in turn promotes the well-being of the entire family.

## Introduction

Parents of infants undergoing staged palliation for hypoplastic left heart syndrome and related single right ventricle anomalies face numerous stressors significantly worsening their well-being.^1^ Stressors include medical challenges affecting their infant, such as the initial diagnosis, complex surgeries, intensive care stays, and ongoing uncertainty regarding their child’s survival and quality of life.^1,2^ These stressors not only pose immediate challenges but also place parents at heightened risk for mental health difficulties.^1,3–6^

In the Family Adaptation study, an ancillary study to the Paediatric Heart Network Single Ventricle Reconstruction trial, funded by the National Heart, Lung, and Blood Institute, parent well-being was significantly below expected population norms.^1,7^ Parent well-being encompasses mental health, emotional resilience, stress management, and broader aspects of life satisfaction and quality of life.^1,8^ The Family Adaptation study demonstrated that symptoms of anxiety and traumatic stress significantly contributed to diminished parental well-being, highlighting the need to further investigate the prevalence of severe anxiety along with the stress and coping mechanisms and access to resources that influence parents’ overall mental health outcomes.^1,5^

Parental mental health challenges not only affect the parents themselves but also have cascading effects on their ability to care for their child, support the child’s recovery, and maintain family stability.^1,9–12^ Understanding state anxiety—defined as a temporary emotional condition influenced by situational factors—may offer valuable insights into supporting the well-being of parents of infants with CHD during the early postpartum period, particularly throughout the critical phases of CHD repair and hospitalisation.^1,5,13^ While anxiety is often a normal and transient reaction to stress, it can become problematic when pervasive or excessive, contributing to conditions such as generalised anxiety disorder, panic disorder, post-traumatic stress disorder, and depressive disorders.^14^

The primary aim of this study was to determine the prevalence of moderate-to-high parental-state anxiety at three key timepoints: following the Norwood procedure, after Stage II surgery, and at a final study visit when the child was approximately 14 months of age. Secondary aims included the identification of differences in state anxiety between mothers and fathers, comparison to norms, and the identification of predictors of elevated parental state anxiety. We hypothesised that greater stress exposure (e.g., parenting a child with a serious illness and family life stress) would be associated with higher parental anxiety. In contrast, psychosocial factors such as adaptive coping strategies (e.g., problem-focused coping, absence of maladaptive coping), social support, and financial security were expected to be associated with lower anxiety. Increased satisfaction with quality of life was hypothesised to be protective against elevated anxiety. Greater understanding of parental anxiety and its associated factors by healthcare providers can ultimately improve long-term mental health outcomes for parents and potentially overall outcomes for the child and family unit.

## Materials and methods

### Design of the study

The Family Adaptation study was an ancillary study to the Pediatric Heart Network Single Ventricle Reconstruction trial, which enrolled 555 infants to receive either a Blalock-Taussig-Thomas or a right ventricle to pulmonary artery shunt during their Norwood procedure.^1,7^ Eleven sites participated in the Family Adaptation study, which was approved by the Institutional Review Board at each site, with all parents providing written informed consent. The Family Adaptation study focused on parents’ perceptions of family function, quality of life, and well-being after their infant underwent the Norwood procedure.^1^ Parents completed 13 self-reported questionnaires at three timepoints: post-Norwood, post-Stage II, and at a final visit when their child was a median age of 14 months.^1,7^

### Participants

Parents who were fluent in English were eligible to participate. Both mothers and fathers were invited to complete individual surveys. Each parent had the opportunity to provide their responses separately. In some families, both parents completed the survey, while in others, only one parent (either the mother or the father) participated. Parents could begin participation at any survey timepoint, and missing a survey did not affect eligibility for subsequent surveys. Parents whose infants died or required heart transplantation were not approached for study participation or were automatically withdrawn if previously enrolled. Data collected prior to the infant’s death or heart transplantation were included in the analysis.

### Measures

Survey measures were organised into booklets, and each participant was invited to complete one booklet per timepoint. All surveys are described in detail, including access instructions, in Supplementary Table 1. Demographic information including age, sex, race, and ethnicity was collected for all participants.

### State-trait anxiety index

The state-trait anxiety index measures symptoms of anxiety and consists of two subscales: (1) STAI-S: state anxiety—temporary feelings of anxiety influenced by specific situations; and (2) STAI-T: trait anxiety—a stable disposition towards anxiety.^13^ The study analysed STAI-S scores and examined how stress factors, psychosocial factors, and quality of life influence STAI-S to better understand parents’ situational anxiety throughout the illness journey. STAI-S scores range from 20 to 80, with higher scores indicating greater state anxiety (none/mild: 20–37; moderate: 38–44; severe: 45–80).

### Inventory of life changes

The inventory of life changes measures cumulative stress resulting from 43 major life events such as divorce, death, or a job change.^15^ Higher scores reflect greater life changes associated with stress, with scores ≥300 indicating a significantly elevated risk of poor health outcomes as a result of stress-induced physical and mental illness.^15^

### Paediatric inventory for parents

The paediatric inventory for parents measures the stress experienced by parents in the context of parenting a child with severe illness.^16^ The instrument evaluates both the frequency and perceived difficulty of stressors across four domains: (1) communication: e.g., “speaking with doctor”; (2) emotional distress: e.g., “feeling scared that my child could get very sick or die”; (3) medical care: e.g., “making decisions about medical care or medicines”; and (4) role function: e.g., “trying to attend to the needs of other family members.”^16^ Higher frequency subscale scores indicate that stressors related to parenting a child with severe illness occur more often, while higher difficulty subscale scores reflect a greater perceived intensity of these stressors.

### Impact on family

The impact on family measures the overall stress experienced by the family as a result of managing a child’s chronic illness within the social and family system.^17^ Example item includes “Our family gives up things because of my child’s illness.”^17^

### Coping health inventory for parents

The coping health inventory for parents measures what parents find helpful to the management of family life when a member of the family is ill, and it has three subscales: (1) family integration, cooperation, and definition of the situation, e.g., “believing that my child will get better”; (2) maintaining social support, self-esteem, and psychological stability, e.g., “getting away by myself”; and (3) understanding the health care situation through communication and consultation, e.g., “talking with the medical staff.”^18^ Higher scores on the coping health inventory for parents indicate parents perceive the elements within each subscale as more helpful.^18^

### Inventory of parent’s experiences

The inventory of parent’s experiences measures parent satisfaction with social support networks, family, and their parenting role.^19^ There are four subscales as follows: (1) Parental role satisfaction: e.g., “Your amount of household responsibility”; (2) Community satisfaction: e.g., “Your involvement in your neighborhood”; (3) Friendship satisfaction: e.g., “The availability of someone to share honestly with you when you are upset or angry”; (4) Family satisfaction: e.g., “The amount of help family members provide.”^19^ Higher scores on the inventory of parent’s experiences indicate parents feel greater satisfaction with the social support domains.^19^

### Family hardiness index

The family hardiness index measures a family’s ability to withstand and adapt to stressful situations, maintain cohesion, and remain resilient.^20^ There are three subscales as follows: (1) commitment: e.g., “We work together to solve problems”; (2) Challenge: e.g., “We seem to encourage each other to try new things and experiences”; and (3) Control: e.g., “we realize our lives are controlled by accidents and luck”—*reverse scored*.^20^ Higher scores on the family hardiness index indicate that a parent perceives stronger levels of commitment, challenge, and control in the context of managing family life.^20^

### Family inventory of resources for management

The family inventory of resources for management assesses parents’ resources for stress management and includes four subscales: (1) esteem and communication: e.g., “In our family we understand what help we can expect from each other”; (2) mastery and health: e.g., “It is hard to get family members to cooperate with each other”; (3) extended family social support: e.g., “Our relatives are willing to listen to our problems”; and (4) financial well-being: e.g., “We seem to have little or no trouble paying our bills on time.”^21^ Higher scores on the family inventory of resources for management indicate that parents perceive their family as having the skills and resources to effectively manage stress.^21^

### Perceived quality of life

The perceived quality of life measures an individual’s degree of satisfaction with their physical, social, and cognitive health and their happiness.^22^ The measure includes four subscales: (1) physical health, e.g., “The amount of walking you do”; (2) social health, e.g., “How respected you are by others”; (3) cognitive health, e.g., “How well you think and remember”; and (4) happiness: e.g., “How happy you are.”^22^

### Statistical analysis

All analyses were conducted using Microsoft Excel (Microsoft Corporation, Redmond, WA), with *p*-values < 0.05 considered statistically significant. Continuous variables are presented as means ± standard deviations or medians (IQR), while categorical variables are expressed as frequencies (counts) and percentages. Variables were assessed visually for normality using histograms and skew. Mean ± standard deviations were reported for normally distributed data, while median (IQR) was used for skewed data.

To analyse differences between mothers and fathers, two-tailed independent *t*-tests were conducted on subdomain stress, psychosocial, and quality of life measures by timepoint. To assess differences in STAI-S between parents in the same family, one-tailed paired *t*-tests were used, and linear regression was used to test for a relationship between partners’ scores. Finally, two-tailed *z*-tests were used to compare mean STAI-S, stress, psychosocial, and quality of life measures scores to published normative or comparator data, separately for mothers and fathers, by timepoint.

Bivariate linear regression models were used to evaluate the relationships between parent STAI-S scores (dependent variable) and stress, psychosocial, and quality of life subscale scores (independent variables). Analysis was performed by timepoint, separately for mothers and fathers. Missing data was handled using a complete-case analysis approach, with the proportion of missing data assessed and found to be low. The overall sample size remained sufficient to ensure statistical reliability. The coefficient of determination (*R* ^2^) was calculated for each bivariate linear model to quantify the proportion of variance in the STAI-S outcome variable explained by the independent stress, psychosocial, or quality of life variables. *R* ^2^ range interpretation was 0.00 ≤ 0.10: very weak relationship; 0.10 ≤ 0.30: weak relationship; and 0.30 ≤ 0.50: moderate relationship.

## Results

### Participant demographic information

Participants included 215 parents (143 mothers and 72 fathers) representing 146 infants. Approximately seventy per cent of CHD was diagnosed prenatally, and most of the pregnancies were planned (Table 1). Maternal median age was 28.7 years (IQR: 24.4–32.4), and paternal median age was 29.7 years (IQR: 26.4–34.6) at the time of their infant’s birth. The median birth order was 2 (IQR: 1–2), and parents reported knowing their spouse or partner for a median of 7 years (IQR: 4–11).

**Table 1.** Demographic variables for mothers, fathers, and infants in the Family Adaptation study. Values are presented as *n*/*N* (%). *N* represents the total number of participants available for each group (mothers, fathers, or infants) at each timepoint. n indicates the number of observed cases for the specific variable. Survey completion 1–7 days prior to discharge. Long LOS in the Norwood group skews post-Norwood age at survey

Maternal characteristics	Post-Norwood	Post-stage II	Final visit
Age at survey in years, *M* (IQR)	29.4 (25.2–32.3)	28.7 (25.3–32.5)	31.1 (27.0–35.4)
Marital Status			
Single, *n*/*N* (%)	13/103 (12)	10/74 (13)	9/67 (13)
Married, *n*/*N* (%)	75/103 (73)	53/74 (72)	53/67 (79)
Living together, *n*/*N* (%)	11/103 (11)	8/74 (11)	2/67 (3)
Divorced, *n*/*N* (%)	3/103 (3)	1/74 (1)	2/67 (3)
Not provided, *n*/*N* (%)	1/103 (1)	2/74 (3)	1/67 (2)
Education			
Less than high school, *n*/*N* (%)	7/103 (7)	4/74 (6)	3/67 (5)
High school graduate, *n*/*N* (%)	13/103 (13)	12/74 (16)	5/67 (7)
Some college or certification, *n*/*N* (%)	30/103 (29)	18/74 (24)	20/67 (30)
College graduate, *n*/*N* (%)	27/103 (26)	21/74 (28)	22/67 (33)
Graduate or professional degree, *n*/*N* (%)	26/103 (25)	19/74 (26)	17/67 (25)

Paternal characteristics	Post-Norwood	Post-stage II	Final visit
Age at survey in years, *M* (IQR)	30.2 (27.9–35.6)	30.8 (26.1–35.6)	32.9 (29.2–37.0)
Marital status			
Single, *n*/*N* (%)	2/49 (4)	3/37 (8)	0/28 (0)
Married, *n*/*N* (%)	38/49 (78)	29/37 (78)	25/28 (89)
Living together, *n*/*N* (%)	7/49 (14)	4/37 (11)	1/28 (4)
Divorced, *n*/*N* (%)	2/49 (4)	1/37 (3)	1/28 (4)
Not provided, *n*/*N* (%)	0/49 (0)	0/37 (0)	1/28 (4)
Education			
Less than high school, *n*/*N* (%)	1/49 (2)	0/37 (0)	0/28 (0)
High school graduate, *n*/*N* (%)	9/49 (18)	9/37 (24)	1/28 (4)
Some college or certification, *n*/*N* (%)	17/49 (35)	12/37 (32)	11/28 (39)
College graduate, *n*/*N* (%)	16/49 (33)	11/37 (30)	9/28 (32)
Graduate or professional degree, *n*/*N* (%)	6/49 (12)	5/37 (14)	7/28 (25)

Infant characteristics	Post-Norwood	Post-stage II	Final visit
Age at survey in days, *M* (IQR)	24 (16–42)	157 (124–201)	432 (411–508)
Gestational age in weeks, μ ± sd	38.3 ± 1.4	38.5 ± 1.4	38.6 ± 1.4
Birth weight in grams, μ ± sd	3095.5 ± 474.8	3097.0 ± 526.4	3195.4 ± 496.0
Planned pregnancy, *n*/*N* (%)	60/107 (56)	45/76 (59)	43/69 (62)
Prenatal diagnosis, *n*/*N* (%)	74/107 (69)	52/76 (68)	49/69 (71)
Male, *n*/*N* (%)	63/107 (59)	49/76 (64)	43/69 (62)
White, *n*/*N* (%)	77/107 (72)	58/76 (76)	53/69 (77)
Black or African American, *n*/*N* (%)	11/107 (10)	4/76 (5)	7/69 (10)
Asian, *n*/*N* (%)	3/107 (3)	1/76 (1)	1/69 (1)
American Indian or Alaska Native, *n*/*N* (%)	1/107 (1)	2/76 (3)	1/69 (1)
Two or more races, *n*/*N* (%)	2/107 (2)	1/76 (1)	1/69 (1)
Some other race, *n*/*N* (%)	5/107 (5)	4/76 (5)	3/69 (4)
Not provided, *n*/*N* (%)	7/107 (7)	6/76 (8)	3/69 (4)
Hispanic or Latino, *n*/*N* (%)	13/107 (12)	8/76 (11)	9/69 (13)
Not Hispanic or Latino, *n*/*N* (%)	86/107 (80)	61/76 (80)	56/69 (81)
Not provided, *n*/*N* (%)	8/107 (8)	7/76 (9)	4/69 (6)

The majority of infants, 90.4%, had hypoplastic left heart syndrome, and 9.6% had other single right ventricle malformations. Infants underwent the Norwood procedure at a median age of 6 days (IQR: 5–8) with 56.1% receiving a right ventricle to pulmonary artery conduit and 43.9% receiving a Blalock-Taussig-Thomas shunt as part of their Norwood operation. Four infants (2.7%) underwent heart transplant during the study (median age: 203 days; IQR: 193–301), with two following the Norwood and two after the Stage II procedure. Eleven infants (7.5%) died (median age: 97 days; IQR: 82–134), including one who had received a heart transplant. Of these deaths, ten occurred after the Norwood procedure and one occurred after the Stage II procedure.

Parents completed the post-Norwood visit when infants were at a median age of 24 days (IQR: 16–42). Post-Stage II surveys were completed when infants had a median age of 157 days (IQR: 124–201), and the final visit surveys were completed when the child was a median age of 432 days (IQR: 411–508). Data from 69 families (47%) represented both mother’s and father’s perspectives for at least one timepoint, as both parents completed surveys. Data from 74 families (51%) represented the mothers’ perspective only, while data from 3 families (2%) represented the fathers’ perspective only.

### Parental STAI-S scores and severity categories

Mothers’ mean state anxiety scores were significantly elevated at all timepoints: 47.7 ± 13.2 post-Norwood, 45.9 ± 12.1 post-Stage II, and 42.1 ± 13.0 at the final study visit (North American Female Norm: 37.6 ± 11.8; *p* < 0.05 for all timepoints; Table 2).^13^ Fathers’ mean state anxiety scores were significantly elevated post-Norwood and post-Stage II (43.5 ± 11.8 and 42.3 ± 13.0, respectively) compared to the North American male norm (36.9 ± 9.1; *p* < 0.05 for both), but not at the final visit (39.0 ± 9.6; *p* = 0.22; Table 2).^13^ Mothers’ average state anxiety scores were significantly higher than fathers’ scores post-Norwood (*p* = 0.03), but not post-Stage II (*p* = 0.08) or at the final visit (*p* = 0.14) (Table 2).

**Table 2.** Subscale scores as means ± sd. Higher values for stress variables indicate greater sources of stress. Higher values for psychosocial variables indicate greater satisfaction with or perceived benefit of a coping mechanism or more protective factors. Higher values for quality of life indicate greater satisfaction within the subscale domain. No comparison for ILC, IoF, PQoL. No gender norms for PIP, IPE, and FIRM. 1 values in bold indicate a significant difference (*p* < 0.05) from the comparison group score; an * indicates a significant difference (*p* < 0.05) between mothers versus fathers

Domain	↑/↓	Abbr.	Subscales	Parent	Score post-Norwood	Score post-stage II	Score final visit	Comparison group score	Comparison group
					Mean±sd	Mean±sd	Mean±sd	Mean±sd	
Outcome variable (STAI-S) and stress variables	Higher value is worse	STAI-S	State	Mothers	47.7 ± 13.2*	**45.9 ± 12.1**	**42.1 ± 13.0**	37.9 ± 11.8	*U.S. norm* ^*13*^
				Fathers	43.5 ± 11.8*	**42.3 ± 13.0**	39.0 ± 9.6	36.9 ± 9.1	
		ILC		Mothers	236.2 ± 124.6	243.9 ± 114.7	194.9 ± 109.8	na	
				Fathers	230.7 ± 126.0	228.2 ± 117.7	205.0 ± 110.7	na	
		PIP	Communication frequency	Mothers	27.7 ± 4.9*	26.6 ± 5.0*	21.3 ± 6.8	18.0 ± 6.7	*Parents of children with cancer* ^*16*^
				Fathers	24.7 ± 4.1*	24.0 ± 3.9*	20.3 ± 5.0	18.0 ± 6.7	
			Communication difficulty	Mothers	21.0 ± 7.3*	20.2 ± 6.3	**17.7 ± 5.9**	19.8 ± 7.4	
				Fathers	18.0 ± 4.7*	18.2 ± 4.5	**16.5 ± 4.7**	19.8 ± 7.4	
			Emotional distress frequency	Mothers	50.2 ± 10.4*	**48.7 ± 10.4**	41.7 ± 12.2	39.2 ± 14.6	
				Fathers	45.7 ±10.5*	**44.5 ±10.9**	38.4 ±11.9	39.2 ± 14.6	
			Emotional distress difficulty	Mothers	51.4 ± 11.5*	48.4 ± 11.5*	**43.9 ± 12.8**	48.4 ± 14.5	
				Fathers	45.1 ± 11.1*	42.5 ± 12.6*	**39.7 ± 12.8**	48.4 ± 14.5	
			Medical care frequency	Mothers	27.1 ± 5.4*	**28.2 ± 5.2**	**23.0 ± 7.6**	16.1 ± 7.1	
				Fathers	24.1 ± 5.7*	**26.3 ± 6.4**	**20.6 ± 7.2**	16.1 ± 7.1	
			Medical care difficulty	Mothers	22.7 ± 6.9*	20.6 ± 7.0	**16.9 ± 6.5**	19.3 ± 7.4	
				Fathers	19.7 ± 4.9*	18.4 ± 6.6	**15.8 ± 4.8**	19.3 ± 7.4	
			Role function frequency	Mothers	32.6 ± 6.6*	30.5 ± 7.4*	**25.0 ± 8.2**	20.6 ± 8.1	
				Fathers	29.5 ± 6.7*	27.0 ± 6.3*	21.7 ± 6.3	20.6 ± 8.1	
			Role function difficulty	Mothers	28.6 ± 7.3*	26.5 ± 7.7*	23.7 ± 8.6*	29.9 ± 9.3	
				Fathers	24.1 ± 7.3*	22.9 ± 7.7*	19.7 ± 5.1*	29.9 ± 9.3	
		IoF		Mothers	37.9 ± 7.3*	37.1 ± 8.0*	32.5 ± 8.0*	na	
				Fathers	33.8 ± 7.0*	34.0 ± 7.5*	31.3 ± 8.7*	na	
Psychosocial variables	Higher value is better	CHIP	Family integration, cooperation, and definition of the situation	Mothers	42.4 ± 7.5	40.2 ± 7.4	40.9 ± 8.4	41.9 ± 7.0	*Parents of children with cardiac illness* ^*18*^
				Fathers	39.8 ± 10.0	39.1 ± 9.6	39.8 ± 8.2	39.8 ± 8.7	
			Social support, self-esteem, psychological stability	Mothers	**26.4 ± 9.3**	**25.7 ± 8.6**	**28.9 ± 10.4**	31.3 ± 7.8	
				Fathers	**24.8 ± 10.1**	**25.9 ± 9.4**	28.9 ± 8.0	28.8 ± 8.1	
			Understanding the health care situation through communication	Mothers	16.1 ± 4.5*	16.3 ± 3.9*	16.6 ± 5.3*	14.9 ± 4.7	
				Fathers	14.0 ± 5.3*	13.7 ± 5.6*	13.6 ± 4.8*	12.8 ± 6.0	
		IPE	Parental role satisfaction	Mothers	2.23 ± 0.51	**2.07 ± 0.44**	**2.04 ± 0.50**	2.22 ± 0.45	*Normative sample* ^*23*^
				Fathers	**2.39 ± 0.45**	2.12 ± 0.47	2.18 ± 0.45	2.22 ± 0.45	
			Community satisfaction	Mothers	1.85 ± 0.85	**1.68 ± 0.76**	**1.44 ± 0.72**	1.97 ± 0.70	
				Fathers	1.95 ± 0.88	**1.46 ± 0.80**	**1.46 ± 0.80**	1.97 ± 0.70	
			Friendship satisfaction	Mothers	2.40 ± 0.62	**2.26 ± 0.60**	**2.21 ± 0.58**	2.42 ± 0.60	
				Fathers	2.34 ± 0.64	2.25 ± 0.57	2.22 ± 0.61	2.42 ± 0.60	
			Family satisfaction	Mothers	**2.50 ± 0.64**	2.25 ± 0.71	2.21 ± 0.59	2.34 ± 0.64	
				Fathers	2.48 ± 0.54	2.22 ± 0.59	2.36 ± 0.62	2.34 ± 0.64	
		FHI	Commitment	Mothers	19.7 ± 3.4	19.0 ± 3.0	19.1 ± 3.8	19.6 ± 3.3	*Parents of children with cardiac illness* ^*20*^
				Fathers	19.4 ± 3.8	19.1 ± 3.2	18.6 ± 3.2	19.1 ± 3.4	
			Challenge	Mothers	12.2 ± 3.3	11.9 ± 2.9	12.0 ± 3.7	12.3 ± 2.8	
				Fathers	12.3 ± 2.5	11.6 ± 3.1	11.4 ± 3.3	11.9 ± 2.7	
			Control	Mothers	**13.8 ± 2.8**	**13.9 ± 2.7**	**14.5 ± 2.7**	13.2 ± 2.9	
				Fathers	**13.9 ± 2.2**	**14.1 ± 2.7**	13.5 ± 3.3	12.8 ± 3.2	
		FIRM	Esteem & communication	Mothers	34.8 ± 6.6	34.9 ± 5.1	**33.0 ± 6.5**	35.0 ± 6.0	*Normative sample* ^*21*^
				Fathers	35.6 ± 7.0	33.6 ± 7.4	33.0 ± 8.7	35.0 ± 6.0	
			Mastery & health	Mothers	38.3 ± 9.5*	37.4 ± 8.5*	36.9 ± 11.4	39.0 ± 9.0	
				Fathers	41.8 ± 7.9*	40.9 ± 8.8*	41.1 ± 10.0	39.0 ± 9.0	
			Extended family social support	Mothers	**9.7 ± 2.1**	**9.5 ± 2.0**	**9.5 ± 2.0**	9.0 ± 2.0	
				Fathers	9.5 ± 2.0	8.8 ± 2.5	9.2 ± 2.4	9.0 ± 2.0	
			Financial well-being	Mothers	28.6 ± 10.1*	29.7 ± 8.5	27.9 ± 10.0	29.0 ± 9.0	
				Fathers	32.6 ± 8.0*	29.5 ± 9.2	30.1 ± 9.7	29.0 ± 9.0	
Quality of life	Higher value is better	PQoL	Physical health	Mothers	5.2 ± 1.9*	4.7 ± 1.9	5.1 ± 1.8	na	
				Fathers	6.1 ± 2.0*	5.3 ± 2.1	5.3 ± 1.7	na	
			Social health	Mothers	5.8 ± 1.6*	5.8 ± 1.6	5.8 ± 1.6	na	
				Fathers	6.5 ± 1.6*	5.9 ± 1.8	5.8 ± 1.6	na	
			Cognitive health	Mothers	6.6 ± 2.2*	6.8 ± 2.0	6.7 ± 2.3	na	
				Fathers	7.3 ± 2.0*	6.6 ± 2.0	6.5 ± 2.5	na	
			Happiness	Mothers	6.3 ± 2.5	6.2 ± 2.3	6.2 ± 2.6	na	
				Fathers	7.0 ± 2.6	6.5 ± 2.4	7.1 ± 2.2	na	

Bold values indicate significantly different from the comparison group.

**Figure 1.**
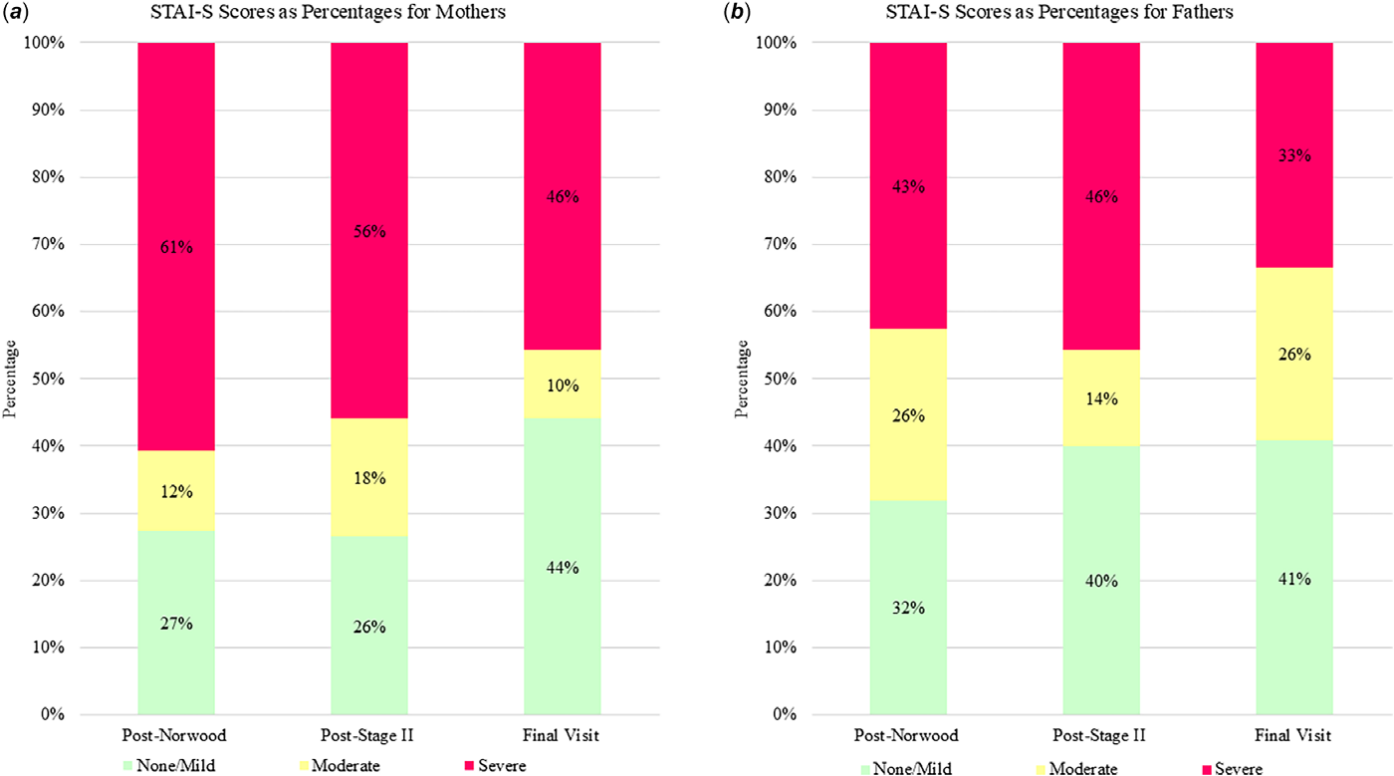
(***a***) The bar graph illustrates the percentage of mothers with none/mild, moderate, and severe symptoms of anxiety at three timepoints. A majority, 61%, of mothers reported severe symptoms of anxiety post-Norwood (infant median age: 24 days; IQR: 16–42). The proportion of mothers with severe symptoms lessens over time, with severe symptoms reported by 56% of mothers post-Stage II (infant median age 157 days; IQR: 124–201) and 46% of mothers at the final visit (child median age 432 days; IQR 411–508). (***b***) The bar graph illustrates the percentage of fathers with none/mild, moderate, and severe symptoms of anxiety at three timepoints. Severe symptoms were reported in 43% of fathers post-Norwood, 46% of fathers post-Stage II and 33% of fathers by the final visit.

Post-Norwood, a substantial proportion of parents fell into a high-risk category of anxiety, with 61% of mothers and 43% of fathers reporting severe symptoms (Figure 1 *(**a**)* and *(**b**)*). At the time of the final visit, 46% of mothers and 33% of fathers reported severe symptoms of anxiety (Figure 1 *(**a**)* and *(**b**)*). Of the 68 mothers who completed more than one STAI-S assessment, close to half (43%) continued to report scores in the severe range across timepoints, indicating a pattern of persistent severe anxiety. For mothers with fluctuating patterns, 19% of mothers showed improvement (i.e., non-severe scores upon repeat assessment), and 12% experienced worsening symptoms (i.e., non-severe scores elevated to severe upon repeat assessment). A subset of mothers, 26%, had scores consistently in the range of no clinical concern. Of the 29 fathers who had completed more than one STAI-S measure, 21% reported scores in the severe range across all timepoints. For fathers with fluctuating patterns of response, 28% of fathers showed improvement (i.e., a non-severe score upon repeat measure), and 14% experienced worsening anxiety over time (i.e., non-severe scores elevated to severe upon reassessment). A subset of fathers, 38%, had scores consistently in the range of no clinical concern.

### STAI-S scores in couples

Paired STAI-S data was available for 66 couples for at least one timepoint (39 couples completed surveys once, 17 twice, and 10 three times). Post-Norwood, married/coupled mothers” mean state anxiety scores were significantly higher than their spouse/partner, 47.3 ± 14.7 versus 43.3 ± 12.2, respectively (*p* < 0.05), but not post-Stage II or at the final visit.

A weak but statistically significant association was observed between partners’ STAI-S scores, indicating that one partner’s anxiety level was modestly related to the others. This association was evident post-Norwood (*R* ^2^ = 0.19, *β* = 0.52, SE = 0.16, *p* < 0.01) and post-Stage II (*R* ^2^ = 0.22, *β* = 0.50, SE = 0.17, *p* < 0.01), but not at the final visit (*R* ^2^ = 0.06, *β* = 0.33, SE = 0.27, *p* = 0.23).

### Inventory of life changes findings

Parents reported sources of stress pertaining to their day-to-day lives, in addition to significant stress related to infants’ illness. Mother’s average inventory of life changes scores did not differ significantly from fathers at any timepoint (Table 2). Approximately 34% (48 of 143) of mothers and 36% (26 of 72) of fathers reported life changes scores greater than or equal to 300, which is a cut-off for increased risk of stress-related physical or mental health illness in the future.^15^ Only mother’s inventory of life changes scores were very weakly associated with STAI-S post-Norwood (*R* ^2^ = 0.08; Table 3), but not at later timepoints, and father’s inventory of life changes scores were never associated with their STAI-S scores.

**Table 3.** Bivariate regression modelling between STAI-S (dependent variable) and stress or psychosocial or quality of life variables (independent variables). *R* ^2^ range interpretation (0.00 < 0.10: very weak relationship; 0.10 < 0.30: weak relationship; 0.30 < 0.50: moderate (mod.) relationship). Stress variables are ILC, PIP, and IoF. Psychosocial variables are CHIP, IPE, FHI, FIRM. The quality of life measure is PQoL. NS, not significant

MOTHERS								
*Visit*		Instrument subscale	*N*	Slope (*β*)	SE	*p*-Value	*R* ^2^	
POST-NORWOOD	PIP	Emotional distress frequency	90	0.90	0.10	*p* < 0.001	0.49	Mod.
	PIP	Emotional distress difficulty	78	0.83	0.10	*p* < 0.001	0.49	
	PIP	Medical care difficulty	68	1.08	0.20	*p* < 0.001	0.31	
	PQoL	Happiness	97	−2.94	0.45	*p* < 0.001	0.31	
	PQoL	Physical health	96	−3.75	0.63	*p* < 0.001	0.28	Weak
	IoF		87	0.94	0.17	*p* < 0.001	0.27	
	PQoL	Social health	85	−4.15	0.75	*p* < 0.001	0.27	
	PIP	Role function difficulty	75	0.88	0.19	*p* < 0.001	0.22	
	PIP	Communication difficulty	73	0.76	0.19	*p* < 0.001	0.19	
	PQoL	Cognitive health	94	−2.64	0.57	*p* < 0.001	0.19	
	PIP	Role function frequency	78	0.88	0.21	*p* < 0.001	0.19	
	PIP	Communication frequency	83	1.13	0.27	*p* < 0.001	0.18	
	FHI	Commitment	96	−1.35	0.37	*p* < 0.001	0.12	
	FIRM	Mastery & health	93	−0.49	0.14	*p* < 0.001	0.12	
	CHIP	Social support, self-esteem, psychological stability	85	−0.47	0.15	*p* < 0.01	0.10	
	IPE	Parental role satisfaction	91	−8.40	2.62	*p* < 0.01	0.10	
	CHIP	Family integration, cooperation, and definition of the situation	87	−0.55	0.19	*p* < 0.01	0.09	Very weak
	ILC		97	0.03	0.01	*p* < 0.01	0.08	
	FIRM	Financial well-being	93	−0.35	0.13	*p* < 0.01	0.07	
	PIP	Medical care frequency	74	0.66	0.28	*p* < 0.05	0.07	
	IPE	Friendship satisfaction	93	−5.67	2.25	*p* < 0.05	0.07	
	IPE	Family satisfaction	95	−4.94	2.17	*p* < 0.05	0.05	
	FIRM	Esteem & Communication	90	−0.46	0.21	*p* < 0.05	0.05	
	IPE	Community satisfaction	92	−3.13	1.65	0.06	0.04	NS
	FHI	Control	91	−0.89	0.50	0.08	0.03	
	FIRM	Extended family social support	97	−0.86	0.64	0.18	0.02	
	FHI	Challenge	95	−0.57	0.43	0.19	0.02	
	CHIP	Understanding the health care situation through communication	92	−0.31	0.31	0.32	0.01	
POST-STAGE II	PIP	Emotional distress frequency	61	0.76	0.12	*p* < 0.001	0.40	Mod.
	PIP	Emotional distress difficulty	56	0.53	0.12	*p* < 0.001	0.26	Weak
	PIP	Role function frequency	60	0.74	0.19	*p* < 0.001	0.20	
	IoF		67	0.62	0.17	*p* < 0.001	0.17	
	PQoL	Social health	59	−3.29	1.02	*p* < 0.01	0.15	
	FIRM	Mastery & health	64	−0.53	0.16	*p* < 0.01	0.15	
	PQoL	Happiness	69	−1.94	0.59	*p* < 0.01	0.14	
	FIRM	Esteem & communication	63	−0.86	0.28	*p* < 0.01	0.14	
	PIP	Role function difficulty	55	0.55	0.21	*p* < 0.01	0.12	
	PIP	Communication frequency	57	0.85	0.31	*p* < 0.01	0.12	
	FHI	Control	67	−1.55	0.55	*p* < 0.01	0.11	
	PQoL	Cognitive health	69	−1.88	0.68	*p* < 0.01	0.10	
	PIP	Communication difficulty	54	0.62	0.25	*p* < 0.05	0.10	
	PIP	Medical care difficulty	63	0.55	0.21	*p* < 0.05	0.10	Very weak
	FIRM	Financial well-being	65	−0.41	0.18	*p* < 0.05	0.08	
	FHI	Commitment	67	−1.14	0.49	*p* < 0.05	0.08	
	PQoL	Physical health	69	−1.76	0.75	*p* < 0.05	0.08	
	PIP	Medical care frequency	64	0.60	0.29	*p* < 0.05	0.07	
	IPE	Family satisfaction	69	−4.38	2.07	*p* < 0.05	0.06	
	FHI	Challenge	67	−1.03	0.50	*p* < 0.05	0.06	
	CHIP	Social support, self-esteem, psychological stability	66	−0.34	0.17	*p* < 0.05	0.06	
	CHIP	Family integration, cooperation, and definition of the situation	54	−0.27	0.20	0.18	0.03	NS
	IPE	Parental role satisfaction	64	−4.89	3.72	0.19	0.03	
	FIRM	Extended family social support	68	−0.79	0.72	0.28	0.02	
	ILC		69	0.01	0.01	0.39	0.01	
	IPE	Community satisfaction	66	−1.64	1.98	0.41	0.01	
	IPE	Friendship satisfaction	68	−1.70	2.47	0.50	0.01	
	CHIP	Understanding the health care situation through communication	65	−0.16	0.39	0.69	0.00	
FINAL VISIT	PIP	Medical care difficulty	50	1.02	0.26	*p* < 0.001	0.25	Weak
	IPE	Parental role satisfaction	57	−13.34	3.21	*p* < 0.001	0.24	
	PIP	Emotional distress difficulty	48	0.50	0.14	*p* < 0.001	0.22	
	PIP	Emotional distress frequency	56	0.49	0.13	*p* < 0.001	0.21	
	PQoL	Social health	55	−3.70	1.02	*p* < 0.001	0.20	
	PQoL	Physical health	57	−3.32	0.92	*p* < 0.001	0.19	
	FIRM	Esteem & communication	58	−0.85	0.24	*p* < 0.001	0.19	
	FHI	Commitment	58	−1.41	0.41	*p* < 0.001	0.18	
	CHIP	Family integration, cooperation, and definition of the situation	57	−0.59	0.19	*p* < 0.01	0.15	
	PQoL	Happiness	58	−1.82	0.60	*p* < 0.01	0.14	
	FIRM	Mastery & health	56	−0.42	0.14	*p* < 0.01	0.14	
	FHI	Control	69	−1.75	0.58	*p* < 0.01	0.14	
	FIRM	Extended family social support	57	−2.38	0.82	*p* < 0.01	0.13	
	IPE	Friendship satisfaction	59	−8.33	2.94	*p* < 0.01	0.12	
	PIP	Role function frequency	49	0.57	0.22	*p* < 0.05	0.12	
	PIP	Role function difficulty	44	0.54	0.23	*p* < 0.05	0.12	
	CHIP	Social support, self-esteem, psychological stability	54	−0.42	0.17	*p* < 0.05	0.11	
	FHI	Challenge	59	−1.13	0.45	*p* < 0.05	0.10	
	PIP	Communication frequency	56	0.60	0.25	*p* < 0.05	0.10	Very weak
	IoF		58	0.49	0.20	*p* < 0.05	0.10	
	PQoL	Cognitive health	57	−1.74	0.72	*p* < 0.05	0.10	
	FIRM	Financial well-being	57	−0.39	0.17	*p* < 0.05	0.09	
	PIP	Communication difficulty	53	0.66	0.30	*p* < 0.05	0.08	
	PIP	Medical care frequency	53	0.46	0.23	*p* < 0.05	0.07	
	CHIP	Understanding the health care situation through communication	58	−0.40	0.33	0.22	0.03	NS
	IPE	Family satisfaction	59	−3.36	3.00	0.27	0.02	
	ILC		59	0.01	0.02	0.36	0.01	
	IPE	Community satisfaction	58	−1.72	2.44	0.48	0.01	

FATHERS								
*Visit*		Instrument subscale	*N*	Slope (*β*)	SE	*p*-Value	*R* ^2^	
POST-NORWOOD	PQoL	Happiness	48	−3.03	0.56	*p* < 0.001	0.39	Mod.
	FHI	Commitment	46	−1.67	0.40	*p* < 0.001	0.29	Weak
	PIP	Emotional distress frequency	44	0.58	0.15	*p* < 0.001	0.27	
	FIRM	Mastery & health	45	−0.72	0.20	*p* < 0.001	0.23	
	FHI	Control	47	−2.49	0.72	*p* < 0.001	0.21	
	PQoL	Physical health	47	−2.46	0.75	*p* < 0.01	0.19	
	FHI	Challenge	48	−2.02	0.63	*p* < 0.01	0.18	
	FIRM	Financial well-being	46	−0.62	0.20	*p* < 0.01	0.17	
	IoF		45	0.71	0.24	*p* < 0.01	0.17	
	PIP	Medical care difficulty	38	0.99	0.38	*p* < 0.05	0.16	
	PIP	Emotional distress difficulty	40	0.41	0.16	*p* < 0.05	0.15	
	PQoL	Social health	47	−2.74	1.07	*p* < 0.05	0.13	
	CHIP	Understanding the health care situation through communication	48	−0.78	0.30	*p* < 0.05	0.13	
	CHIP	Family integration, cooperation, and definition of the situation	44	−0.41	0.17	*p* < 0.05	0.13	
	IPE	Friendship satisfaction	47	−6.74	2.77	*p* < 0.05	0.12	
	PIP	Communication frequency	44	0.95	0.41	*p* < 0.05	0.11	
	PIP	Role function difficulty	43	0.55	0.24	*p* < 0.05	0.11	
	PIP	Role function frequency	45	0.57	0.25	*p* < 0.05	0.11	
	PQoL	Cognitive health	48	−1.89	0.81	*p* < 0.05	0.11	
	FIRM	Esteem & communication	46	−0.51	0.23	*p* < 0.05	0.10	Very weak
	IPE	Parental role satisfaction	47	−8.35	3.90	*p* < 0.05	0.09	
	PIP	Communication difficulty	40	0.67	0.39	0.10	0.07	NS
	FIRM	Extended family social support	47	−1.59	0.86	0.07	0.07	
	IPE	Family satisfaction	48	−5.57	3.07	0.08	0.07	
	PIP	Medical care frequency	39	0.46	0.33	0.17	0.05	
	CHIP	Social support, self-esteem, psychological stability	46	−0.12	0.18	0.49	0.01	
	ILC		48	0.00	0.01	0.81	0.00	
	IPE	Community satisfaction	45	0.38	2.13	0.86	0.00	
POST-STAGE II	PQoL	Happiness	35	−3.82	0.66	*p* < 0.001	0.51	Mod.
	PQoL	social health	34	−4.89	0.94	*p* < 0.001	0.46	
	PIP	Emotional distress difficulty	30	0.66	0.15	*p* < 0.001	0.40	
	PIP	Emotional distress frequency	33	0.73	0.17	*p* < 0.001	0.37	
	PQoL	Physical health	35	−3.54	0.90	*p* < 0.001	0.32	
	FHI	Challenge	35	−2.13	0.62	*p* < 0.01	0.26	Weak
	CHIP	Social support, self-esteem, psychological stability	35	−0.69	0.21	*p* < 0.01	0.25	
	PIP	Communication difficulty	32	1.40	0.46	*p* < 0.01	0.24	
	FHI	Commitment	35	−1.95	0.62	*p* < 0.01	0.23	
	FIRM	Mastery & health	33	−0.72	0.24	*p* < 0.01	0.22	
	PQoL	Cognitive health	35	−2.97	0.99	*p* < 0.01	0.21	
	PIP	Medical care difficulty	32	0.97	0.35	*p* < 0.01	0.21	
	PIP	Role function difficulty	33	0.77	0.28	*p* < 0.01	0.20	
	FIRM	Esteem & communication	35	−0.76	0.27	*p* < 0.01	0.19	
	IPE	Parental role satisfaction	35	−11.35	4.91	*p* < 0.05	0.14	
	IoF		35	0.62	0.28	*p* < 0.05	0.13	
	PIP	Communication frequency	34	1.16	0.56	*p* < 0.05	0.12	
	FIRM	Extended family social support	35	−1.78	0.85	*p* < 0.05	0.12	
	CHIP	Family integration, cooperation, and definition of the situation	35	−0.45	0.22	*p* < 0.05	0.11	
	PIP	Role function frequency	34	0.69	0.34	*p* < 0.05	0.11	
	IPE	Friendship satisfaction	35	−7.56	3.88	0.06	0.10	NS
	CHIP	Understanding the health care situation through communication	35	−0.71	0.38	0.07	0.10	
	FHI	Control	34	−1.50	0.85	0.09	0.09	
	IPE	Family satisfaction	33	−6.76	3.98	0.10	0.09	
	IPE	Community satisfaction	35	−4.67	2.83	0.11	0.08	
	FIRM	Financial well-being	35	−0.33	0.24	0.18	0.05	
	PIP	Medical care frequency	35	0.28	0.35	0.43	0.02	
	ILC		35	0.01	0.02	0.54	0.01	
FINAL VISIT	FIRM	Mastery & health	22	−0.60	0.16	*p* < 0.001	0.40	Mod.
	IPE	Parental role satisfaction	25	−12.13	3.49	*p* < 0.01	0.34	
	FHI	Challenge	27	−1.60	0.51	*p* < 0.01	0.28	Weak
	FHI	Commitment	27	−1.51	0.52	*p* < 0.01	0.25	
	PQoL	Social health	27	−3.58	1.24	*p* < 0.01	0.25	
	PQoL	Happiness	27	−2.67	0.96	*p* < 0.01	0.24	
	PIP	Communication difficulty	23	0.94	0.42	*p* < 0.05	0.19	
	CHIP	Understanding the health care situation through communication	26	−0.79	0.35	*p* < 0.05	0.18	
	FIRM	Esteem & Communication	26	−0.46	0.22	*p* < 0.05	0.16	
	IoF		25	0.44	0.22	0.06	0.15	NS
	PIP	Role function difficulty	21	0.60	0.34	0.09	0.14	
	CHIP	Family integration, cooperation, and definition of the situation	25	−0.47	0.24	0.07	0.14	
	IPE	Friendship satisfaction	25	−6.24	3.35	0.07	0.13	
	FIRM	Extended family social support	26	−1.48	0.82	0.08	0.12	
	PQoL	Cognitive health	27	−1.38	0.80	0.09	0.11	
	FHI	Control	27	−0.94	0.55	0.10	0.11	
	PIP	Emotional distress difficulty	21	0.25	0.17	0.15	0.10	
	PIP	Emotional distress frequency	26	0.24	0.16	0.14	0.09	
	CHIP	Social support, self-esteem, psychological stability	23	−0.31	0.26	0.24	0.06	
	ILC		27	0.02	0.02	0.24	0.06	
	PIP	Role function frequency	23	0.31	0.29	0.29	0.05	
	PIP	Medical care difficulty	23	0.40	0.44	0.38	0.04	
	PQoL	Physical health	27	−1.13	1.19	0.35	0.03	
	PIP	Medical care frequency	24	0.25	0.28	0.39	0.03	
	IPE	Community satisfaction	26	−2.34	2.55	0.37	0.03	
	FIRM	Financial well-being	24	−0.19	0.21	0.39	0.03	
	IPE	Family satisfaction	25	−2.48	3.21	0.45	0.03	
	PIP	Communication frequency	25	0.29	0.40	0.48	0.02	

### Paediatric inventory for parents findings

Post-Norwood, mothers reported significantly higher (worse) communication, emotional distress, medical care, and role function frequency and difficulty subdomain scores compared to fathers (Table 2). Mother’s role function difficulty scores were consistently higher (worse) than fathers across all timepoints. Compared to parents of children with cancer, communication, emotional distress, medical care, and role function frequency subdomain scores were significantly higher, indicating that stressful events pertaining to parenting a child with a serious illness were occurring more frequently for CHD parents (Table 2).^16^

Mothers’ communication, emotional distress, medical care, and role function frequency and difficulty subscale scores were significantly associated with STAI-S at all timepoints, the highest association observed for emotional distress frequency and difficulty post-Norwood (*R* ^2^ = 0.49; Table 3). In comparison, paediatric inventory for parents subdomain scores were less consistently associated with father’s STAI-S scores, with the highest association observed post-Stage II in the subdomain emotional distress difficulty (*R* ^2^ = 0.40; Table 3).

### Impact on family findings

Mothers’ impact on family scores was significantly higher (worse) than fathers’ scores at all timepoints (Table 2). Impact on family was consistently associated with mothers’ STAI-S scores, with the highest association post-Norwood (*R* ^2^ = 0.27; Table 3), while fathers’ impact on family scores was significantly associated with STAI-S only post-Norwood and post-Stage II, with the highest association post-Norwood (*R* ^2^ = 0.17; Table 3).

### Coping health inventory for parents findings

No difference was observed between mothers’ scores versus fathers’ scores for family integration, cooperation, and definition of the situation and social support, self-esteem, and psychological stability (Table 2). Compared to parents of children with cardiac illness (any type), this study population’s social support, self-esteem and psychological stability were significantly worse (Table 2).^18^ Mothers’ understanding of the healthcare situation through communication scores was significantly higher (better) than fathers” and the comparator population” at all timepoints (Table 2).

Mothers’ family integration, cooperation, and definition of the situation subscale scores were significantly associated with STAI-S post-Norwood and at the final visit, with the highest association at the final visit (*R* ^2^ = 0.15; Table 3). Mothers’ social support, self-esteem, and psychological stability subscale scores were significantly associated with STAI-S at all timepoints, with the highest association at the final visit (*R* ^2^ = 0.11; Table 3). Understanding the healthcare situation through communication was not associated with mothers’ STAI-S.

Fathers’ family integration, cooperation, and definition of the situation scores were significantly associated with STAI-S post-Norwood and post-Stage II, the highest post-Norwood (*R* ^2^ = 0.13; Table 3). Fathers’ social support, self-esteem, and psychological stability scores were significantly associated with STAI-S post-Stage II only (*R* ^2^ = 0.25; Table 3). Fathers’ understanding of the health care situation through communication scores was significantly associated with STAI-S post-Norwood and at the final visit, highest at the final visit (*R* ^2^ = 0.18; Table 3).

### Inventory of parent’s experiences findings

Inventory of parent’s experiences subdomain scores did not differ significantly between mothers and fathers (Table 2). Mother’s parental role, community, and friendship scores were significantly lower (worse) than the norm post-Stage II and by final visit, while father’s community scores were significantly lower (worse) than the norm post-Stage II and by final visit (Table 2), but family satisfaction scores were similar to norms.^23^

Mothers’ parental role scores were significantly associated with STAI-S post-Norwood and at final follow-up, highest at the final visit (*R* ^2^ = 0.24; Table 3). Mothers’ community scores were not significantly associated with STAI-S. Mothers’ friendship scores were significantly associated with STAI-S post-Norwood and at the final visit, with the highest association at the final follow-up (*R* ^2^ = 0.12; Table 3). Mothers’ family scores were significantly associated with STAI-S post-Norwood and post-Stage II, highest post-Stage II (*R* ^2^ = 0.06; Table 3).

Fathers’ parental role satisfaction was consistently associated with STAI-S, highest at the final visit (*R* ^2^ = 0.34; Table 3), but otherwise only friendship satisfaction was associated with fathers’ STAI-S post-Norwood (*R* ^2^ = 0.12; Table 3). Fathers’ community and family scores were not significantly associated with STAI-S.

### Family hardiness index findings

Mothers’ mean family hardiness index subscale scores did not differ significantly from fathers (Table 2). Commitment and challenge subscale scores did not differ from the comparison group, but control subscale scores were significantly higher (better) (Table 2).^20^

Mothers’ commitment scores were significantly associated with STAI-S at all timepoints, with the strongest association at the final visit (*R* ^2^ = 0.18; Table 3). Mothers’ challenge scores were significantly associated with STAI-S post-Stage II and at the final visit, with the strongest association at the final visit (*R* ^2^ = 0.10; Table 3). Mothers’ control scores were significantly associated with STAI-S post-Stage II and at the final visit, highest at the final visit (*R* ^2^ = 0.14; Table 3).

Fathers’ commitment scores were significantly associated with STAI-S at all timepoints, highest post-Norwood (*R* ^2^ = 0.29; Table 3). Fathers’ challenge scores were significantly associated with STAI-S at all timepoints, highest at the final visit (*R* ^2^ = 0.28; Table 3). Fathers’ control scores were significantly associated with STAI-S post-Norwood (*R* ^2^ = 0.21; Table 3).

### Family inventory of resources for management findings

Mastery and health scores were significantly lower (worse) for mothers than fathers (Table 2) and the norm (Table 2).^21^ Mothers’ extended family social support was higher (better) than the norm (Table 2).^21^ Fathers’ mastery and health and financial well-being were higher (better) than the norm (Table 2).^21^

Mothers’ esteem and communication scores were significantly associated with STAI-S at all timepoints, highest at the final visit (*R* ^2^ = 0.19; Table 3). Mothers’ mastery and health scores were significantly associated with STAI-S at all timepoints, highest post-Stage II (*R* ^2^ = 0.15; Table 3). Mothers’ extended family scores were significantly associated with STAI-S at the final visit (*R* ^2^ = 0.13; Table 3). Mothers’ financial well-being scores were significantly associated with STAI-S at all timepoints, highest at the final visit (*R* ^2^ = 0.09; Table 3).

Fathers’ esteem and communication scores were significantly associated with STAI-S at all timepoints, highest post-Stage II (*R* ^2^ = 0.19; Table 3). Fathers’ mastery and health scores were significantly associated with STAI-S at all timepoints, with the strongest association at the final visit (*R* ^2^ = 0.40; Table 3). Fathers’ extended family scores were significantly associated with STAI-S post-Stage II (*R* ^2^ = 0.12; Table 3). Fathers’ financial well-being scores were significantly associated with STAI-S post-Norwood (*R* ^2^ = 0.17; Table 3).

### Perceived quality of life findings

Post-Norwood, mothers’ physical, social, and cognitive health scores were significantly lower (worse) than father’s scores (Table 2), but mothers’ scores did not differ from fathers’ scores post-Stage II or at the final visit.

Mothers’ physical, social, and cognitive health and happiness scores were significantly associated with STAI-S at all timepoints, all having strong associations post-Norwood (physical: *R* ^2^ = 0.28; social: *R* ^2^ = 0.27; cognitive: *R* ^2^ = 0.19; happiness: *R* ^2^ = 0.31; Table 3).

Fathers’ physical health scores showed significant associations with STAI-S post-Norwood and post-Stage II, with the strongest correlation post-Stage II (*R* ^2^ = 0.32; Table 3). Fathers’ social health scores were significantly associated with STAI-S at all timepoints, highest post-Stage II (*R* ^2^ = 0.46; Table 3). Fathers’ cognitive health scores were significantly associated with STAI-S post-Norwood and post-Stage II, highest post-Stage II (*R* ^2^ = 0.21; Table 3). Fathers’ happiness scores were significantly associated with STAI-S at all timepoints, highest post-Stage II (*R* ^2^ = 0.51; Table 3).

## Discussion

Parents of infants with single-ventricle CHD who underwent the Norwood procedure reported elevated state anxiety with a substantial proportion falling within the severe range, highlighting the significant emotional toll during this challenging postoperative period. While anxiety levels decreased over time, many parents continued to experience severe symptoms many months after their infant’s surgical procedures, underscoring the need for ongoing emotional support throughout all stages of care. Early screening for anxiety should be integrated into routine clinical care, including prenatal care, and continue longitudinally to monitor changes.^1,5,24–26^ Electronic medical records could facilitate mental health screening of parents by incorporating brief self-reported questionnaires to identify needs, flag concerns, and provide resources.^27,28^ Serial screenings might identify parents at the highest risk for mental health disorders, enabling timely interventions to mitigate the long-term emotional impact of caring for a child with complex CHD.^28^

Our findings highlight notable differences in how stress, psychosocial, and quality of life factors relate to state anxiety among mothers and fathers of children with complex CHDs. A moderate number of parents reported life changes above a threshold that indicated a risk of stress-induced mental and physical illness in the near future.^15^ While there is a growing awareness of the impact of stress on parent mental health, the relationship between stress and physical health outcomes for parents remains less explored. Mothers exhibited worse stress-related scores than fathers, with emotional distress tied to the child’s illness emerging as a consistent predictor of their anxiety across timepoints. Maternal anxiety both before and after childbirth is a risk factor for developing a major depressive disorder.^29^ Unlike mothers, whose anxiety was predominantly linked to emotional distress and stress tied to the experience of parenting a child with serious illness, fathers’ anxiety was more strongly influenced by psychosocial factors. Specifically, satisfaction with the parenting role, commitment, challenge, esteem, and mastery were consistent predictors of father’s STAI-S scores. These findings underscore the need for tailored psychological support that accounts for the distinct stressors and coping mechanisms of each parent. While interventions aimed at reducing emotional distress may be beneficial, particularly for mothers, supporting fathers in fostering a feeling of cohesion and adaptive coping strategies may help mitigate their anxiety.

Parents scored highly on coping strategies related to staying organised, working well with others, and making sense of their situation. Mothers also showed strong understanding of their child’s health care needs—together suggesting an active and engaged approach to coping. Strategies to boost fathers’ understanding of the health care situation through communication could mitigate their anxiety. For parents, lower scores in the area of self-care, esteem, and social support indicate potential areas for improvement. Also, low satisfaction in parent role, community, and friendships suggests challenges in maintaining social connections and feeling adequately supported within their broader social circles, highlighting the need for strategies to strengthen social support and personal well-being.

The challenge of addressing the infant’s medical care and clinical management is paramount; it is also important to consider parent stress and coping, influenced by complex, multifactorial elements. Parental responses and behaviours are shaped by their life experiences, external pressures, and coping abilities on top of the stress related to their child’s medical journey, and some of these factors are not always visible to the healthcare provider. An understanding of the complex interplay of factors is valuable for all providers, especially novice providers, who may struggle to face the challenge of managing the child’s clinical needs while supporting family-clinician interactions.^25,26^ By being aware of patterns in stress and coping in parents, clinicians can better support parents” mental health, fostering a more holistic and empathetic approach to care.

These findings should be interpreted in light of the key strengths and limitations of the study. The use of multiple measures at different timepoints and participation from both mothers and fathers are notable strengths, allowing for a more comprehensive understanding of parental experiences over time. However, all study measures were developed and validated for English-speaking populations, and the perspectives of non-English-speaking individuals are not represented. Selection bias is also a potential limitation in our study, due to both the decision to enrol in the study and differential participation of parents across the three visits. Parents who declined participation may have differed from those who enrolled.

## Conclusion

Many parents of infants undergoing staged palliation for hypoplastic left heart syndrome and related single right ventricle anomalies report moderate to severe anxiety. A child’s health cannot be treated in isolation from their family environment. Research has consistently shown that poor parent mental health, like high anxiety, can significantly affect a parent’s ability to care for an ill child and manage familial responsibilities, influencing the child’s development and overall health.^1,9–12^ Notably, stress and coping factors differed in their predictive value between mothers and fathers, suggesting the need for tailored clinical approaches that account for parent-specific patterns of distress and adaptation. Our findings highlight the importance of recognising parental stress, with notable differences between mothers and fathers in anxiety levels and the distinct stress and psychosocial factors influencing anxiety, as critical elements in the family dynamic. Clinicians are encouraged to assess the mental health of the entire family, identifying parents who may benefit from additional support in managing anxiety and coping. By adopting a holistic approach, healthcare providers can promote better outcomes not only for the child but also for the entire family.

## Supporting information

10.1017/S1047951126111809.sm001 Bainton et al. supplementary material 1 Bainton et al. supplementary material

10.1017/S1047951126111809.sm002 Bainton et al. supplementary material 2 Bainton et al. supplementary material

## Supplementary material

The supplementary material for this article can be found at https://doi.org/10.1017/S1047951126111809.
