# Supplementary material for: Stress, coping, protective factors, and quality of life in parents of infants with CHD: associations with state anxiety
Source: Cardiol Young. 2026 Apr 6;36(3):565–79. doi: 10.1017/S1047951126111809 (PMC13107193; doi:10.1017/S1047951126111809)
Supplement: Bainton et al. supplementary material 1 — Bainton et al. supplementary material [file S1047951126111809sup001.pdf]

Supplementary Table 1. Family Adaptation study instruments and the study instrument subscales. Primary outcome measure (STAI-S, state). Stress variable measures (ILC, PIP, IoF). Psychosocial factors measures (CHIP, IPE, FHI, FIRM). Quality of life measure (PQoL). ILC is also known as the Life Stress Inventory or Social Readjustment Rating Scale.

Instrument and Access	α (alpha)	Direction of the Score	Instrument Subscales	Subscale Items	Subscale Description
State-Trait Anxiety Index (STAI)[13]<br><br>Access: The full measure is available for purchase from the publisher, Mind Garden, Inc.	0.86-0.95	Higher scores indicate greater levels of anxiety (none/mild: 20-37; moderate: 38-44; severe: 45-80).	Trait Anxiety	20-items	Trait anxiety endures meaning it is consistent across various situations.
			State Anxiety	20-items	State anxiety are temporary feelings of anxiety that can fluctuate depending on circumstances.
Inventory of Life Changes (ILC)[15]<br><br>Access: Measure available in original publication and widely available in educational/research repositories for non-commercial use.	0.96–0.89	<150 low stress, low probability of stress-related disorder. 150-299 moderate stress, 50% chance of stress-related disorder. >300 high stress, 80% chance of stress-related disorder.	No subscales	43-items	A measure of life stress based on life changes such as divorce, death, job loss, etc.
Pediatric Inventory for Parents (PIP)[16]<br>Access: Contact original authors/institution for instrument and permission for research use.	0.80-0.96	Higher scores indicate greater levels of stress related to frequency or difficulty of the subscale category.	Communication Frequency	9-items	Communication that pertains to the child's illness that occurs between parent and health care team or other family members.
			Communication Difficulty	9-items	
			Emotional Distress Frequency	15-items	Emotional distress directly related to the caregiving for a child with severe illness.
			Emotional Distress Difficulty	15-items	
			Medical Care Frequency	8-items	Stress related to making decisions about medical care or medicine, helping with medical procedures, and handling child's medical routines.
			Medical Care Difficulty	8-items	
			Role Function Frequency	10-items	Stress related to attending to needs of other family members and work while managing care of a child with serious illness.
			Role Function Difficulty	10-items	
Impact on Family (IoF)[17]<br>Access: Available with permission from the author.	0.87	Higher scores indicate greater negative family impact.	No subscales	15-items	A measure of general impact on the social and family system related to chronic illness in a child.
Coping Health Inventory for Parents (CHIP)[18]<br><br>Access: Contact McCubbin Resilience Center or authors for permission/instrument materials.	0.71-0.79	Higher scores indicate adaptive coping strategies and positive adjustment to stress.	Coping Pattern I	19-items	Family integration, cooperation, and definition of the situation (coping).
			Coping Pattern II	18-items	Maintaining social support, self-esteem, and psychological stability (coping).
			Coping Pattern III	8-items	Understanding the health care situation through communication and consultation (coping).

Inventory of Parent's Experiences (IPE)[19]<br><br>Access: Available from authors for research purposes.	0.74-0.85	Higher scores indicate greater satisfaction in the category of the subscale.	Parental Role Satisfaction	7-items	Household responsibility, time for self, time with child, and time for social activities (coping & protective factors).
			Community Satisfaction	2-items	Involvement and satisfaction with neighborhood and community groups (coping & protective factors).
			Friendship Satisfaction	3-items	Involvement and satisfaction in friend relationships (protective factors).
			Family Satisfaction	3-items	Involvement and satisfaction with family relationships (protective factors).
Family Hardiness Index (FHI)[20]<br><br>Access: Contact McCubbin Resilience Center or authors for permission/instrument materials.	0.82	Higher scores indicate that the parent feels their family possesses the characteristics necessary to thrive in face of adversity and maintain well-being over time.	Commitment	8-items	Family's sense of internal strengths, dependability, and ability to work together (coping & protective factors).
			Challenge	6-items	Family's efforts to be innovative, active, to experience new things and to learn (coping & protective factors).
			Control	6-items	Family's sense of being in control of family life rather than being shaped by outside events and circumstances (maladaptive coping strategies and vulnerabilities).
Family Inventory of Resources for Management (FIRM)[21]<br><br>Access: Contact authors or the McCubbin Resilience Center (measure distribution) for permission and instrument materials.	0.89	Higher scores reflect the presence of protective factors and resources within the family system, which contribute to resilience, well-being, and successful management of stress and adversity.	Esteem & Communication	15-items	Family esteem, communication, mutual assistance, optimism, solving problems, encouragement and autonomy of members (protective factors).
			Mastery & Health	20-items	Mastery over family events and outcomes, family mutuality, physical and emotional health (maladaptive coping strategies and vulnerabilities).
			Extended Family Social Support	4-items	Help and support from relatives (protective factors).
			Financial Well-Being	16-items	Ability to meet financial commitments, adequacy of financial resources, ability to help others, optimism on family's financial future (protective factors).
Perceived Quality of Life (PQoL)[22]<br><br>Access: Contact Seattle Quality of Life Group for permission and instrument materials.	0.88	Higher scores indicate better quality of life.	Physical Health	5-items	Satisfaction with physical health and well-being.
			Social Health	11-items	Satisfaction with social health and well-being.
			Cognitive Health	2-items	Satisfaction with cognitive health and well-being.
			Happiness	1-item	Satisfaction with level of happiness.
